# The role of cattle manure-driven polysaccharide precursors in humus formation during composting of spent mushroom substrate

**DOI:** 10.3389/fmicb.2024.1375808

**Published:** 2024-07-18

**Authors:** Fengjun Yang, Mengmeng Wang, Liqin Zhao, Bowen Fan, Ning Sun, Juncai Liu, Xinying Sun, Ziming Dong

**Affiliations:** College of Horticulture and Landscape Architecture, Heilongjiang Bayi Agricultural University, Daqing, China

**Keywords:** composting, cattle manure, spent mushroom substrate, humification, precursors, core microbes

## Abstract

The study examined the impact of adding cattle manure to the composting process of *Agaricus bisporus* mushroom substrate on compost humification. A control group CK comprised entirely of *Agaricus bisporus* mushroom substrate, while the experimental group CD (70 percent *Agaricus bisporus* mushroom substrate and 30 percent cattle manure) comprised the two composting treatments that were established. The study determined that the addition of cow dung has promoted the formation of humus components. Particularly, humic substance (HS-C) and humic acid (HA) increased by 41.3 and 74.7%, respectively, and the ratio of humic acid to fulvic acid (HA/FA) also increased by 2.78. It showed that the addition of cow dung accelerated the synthesis and decomposition of precursors, such as polysaccharides, polyphenols, and reducing sugars. Thereby promoting the formation of humic acid. Network analysis revealed that adding cow dung promoted microbial interactions increased the complexity and stability of the bacterial and fungal symbiotic network, enhanced cooperation and reciprocity among microbes, and assisted in transforming fulvic acid (FA) components. Structural equation modeling (SEM) is a multivariate data analysis method for analyzing complex relationships among constructs and core indicators. SEM illustrated that introducing cattle manure into the composting process resulted in alterations to the correlation between physicochemical parameters and the microbial community, in addition to humus formation. Polysaccharides are the primary precursors for polymerization to form HA, which is an essential prerequisite for the conversion of fulvic acid to humic acid. Additionally, microbes affected the formation of humus, with bacteria substantially more influential than fungi. These findings provide new ideas for regulating the degree of humification in the composting process and have important practical implications for optimizing mushroom cultivation and composting techniques today.

## 1 Introduction

Humus constitutes the principal component of soil organic matter, presenting itself as a complex polymeric compound derived from various precursors in nature. It forms through a sequence of biochemical reactions under the influence of microbial activity. Meanwhile, humus is a crucial indicator of compost quality due to its ability to keep soil carbon, remediate soil pollution, and promote plant growth (Wei et al., 2022). Furthermore, humic acid (HA) and fulvic acid (FA) are the predominant components of humic substances (HS) and are employed to evaluate the quality of the final compost product. Moreover, the formation of HS typically passes through two phases: the first phase is the generation of HS precursor substances, where microbes decompose organic matter to form HS precursors, including polyphenols, amino acids, polysaccharides, and reducing sugars (Fukuchi et al., 2010; Wu et al., 2017a). Precursors are polymerized in the second phase to produce HS. Microbial action generates HS precursors via diverse mechanisms, one of which is the phenolic protein pathway (Zhang et al., 2018), the lignin protein pathway (Qi et al., 2020), and the Maillard (sugar-amine condensation) pathway (Zhao et al., 2017c).

The cultivation process of edible mushrooms results in a remarkable amount of residues. The annual production of mushroom residues in China surpasses 13 million tons as a result of the expansion of the edible fungi sector (Huang et al., 2018). Besides, the mushroom residue is ample in nitrogen, phosphorus, potassium, and other mineral elements, and is a valuable sustainable resource (Zhang et al., 2014). High-temperature composting of mushroom residue can reduce the amount of plant pathogenic bacteria comprising the residue and enhance its fertilizer efficiency. Nonetheless, due to the high amount of difficult-to-degrade lignocellulose in the mushroom residue, it has low composting efficiency due to its sluggish decomposition during the composting process. Mixed composting can take advantage of each other’s properties to attain superior quality compost (Awasthi et al., 2020). Simultaneously managing distinct types of waste reduces the need for additional disposal investments. Animal manure is a crucial contributor to organic waste pollution, with over 3 billion tons produced annually (Zhou et al., 2014). Its utilization as a co-substrate for composting balances the C/N ratio of the initial compost as well as provides a substantial supplier of nitrogen and effectively degradable organic matter. At present, there is more research on the resource utilization technology of mushroom slag at home and abroad, and the related research results have been applied and achieved good economic, ecological, and social benefits. However, the internal microbial community and nutrients of the bacterial residue to further explore and analyze the application of bacterial residue for soil improvement and explore the mechanism of composting and other aspects of in-depth research is still to be done.

Microbes perform a pivotal role in the degradation and transformation of biomolecules, and microbial activity is critical to the balance between humification and mineralization (Avdalovic et al., 2021). The transformation from degradable organic molecules to stabilized humic acids is based on microbial activity and chemical mechanisms. Flexible adaptation to metabolic environments increases the activity of bacterial communities, which are also intimately connected to the conservation and production of major precursors (Duan et al., 2019a). Fungi play an essential role in the degradation of lignin, converting it and phenols into humic substances (Gu et al., 2022). Concerning that precursors can substantially influence HS formation, identifying key microbial that influence the dynamics of precursors is vital for understanding HS formation. Nonetheless, the composition of key microbial may be affected by the complex environmental conditions of composting (Zhang et al., 2011). Hence, Understanding the formation of humic substances involves establishing the connections between precursor substances, microbial communities, and physicochemical parameters. Numerous studies have been conducted on the decomposition and transformation of HS by microbes. Nonetheless, it remains unclear whether specific microbes are explicitly involved in the formation of HS, further transformed through precursors or both. Additionally, who has a greater role of bacteria and fungi in the formation of humus is yet unclear. The relationship between precursors and key microbes that influence HS formation during the composting of mixed materials has been the subject of fewer studies. Consequently, studying the precursor substances and microbes that promote the production of HS is necessary.

In this study, we investigated the humification effect of cattle manure in mushroom substrate compost. The aim of this study is to (1) Explore the effect of cattle manure on the changes in humus components during composting of mushroom substrate. (2) Exploring the effects of adding cattle manure on microbial communities (3) Identify key factors affecting the compost humification process from multiple perspectives utilizing procrustes analysis, network analysis, and structural equation modeling (SEM). (4) Focuses on the contribution and mechanism of action of microbial communities that are precursors of humic acid formation. This study provides novel ideas for enhancing humic acid content and compost quality for comprehensive resource use.

## 2 Materials and methods

### 2.1 Composting experimental setup and sample collection

The study was conducted at the experimental base of Heilongjiang Bayi Agricultural University. *Agaricus bisporus* substrate was collected from Daqing Hengrui Edible Mushroom Company, and cattle manure was gathered from Anda’s farm. The physicochemical properties of the raw materials (Detailed information is available in Supplementary Table S1). Besides, a strip-stack composting method was employed, with a trapezoidal pile, 2.5 m × 1.5 m at the bottom, and 1.9 m × 0.9 m at the top, with a height of 1.5 m. Through pre-experimentation, a mixture of *Agaricus bisporus* mushroom substrate and cattle manure in a 7:3 (w:w) ratio was determined for the CD treatment; the CK treatment consisted solely of *Agaricus bisporus* mushroom substrate that had been composted. Furthermore, the initial moisture content (MC) was approximately 60%, and pile and ambient temperatures were measured daily during the composting process. By the temperature change on the 10th day, 20th day, the 29th day artificially turn the pile 3 times on the condition that the pile temperature stabilization tends to resemble the surrounding temperature that the compost decomposition is complete. Based on temperature change, the collected samples from the 1st, 3rd, 17th, and 36th days were picked to illustrate the mesophilic, thermophilic, cooling, and maturation phases. A random sampling technique was employed to select five points at 20, 70, and 120 cm from the top of the pile; the resulting samples had an approximate mass of 0.5 kg each. Following mixing, the samples were gathered in two parts, one was stored at −80°C for high-throughput sequencing, the other was air-dried and sieved for the analysis of physicochemical indexes, and the four periods of CK and CD were termed CK1, CK3, CK17, CK36, CD1, CD3, CD17, and CD36.

### 2.2 Determination of the physicochemical properties and humus components

An automatic temperature recorder was used to measure compost temperature and ambient temperature during the composting process, and the temperatures at the top, middle, and bottom of the pile were recorded at 3:00 p.m. each day. Water content (WC) was determined by drying the samples to constant weight in a 105°C oven; Total organic carbon (TOC) was ascertained by the externally heated potassium dichromate-volumetric method and total nitrogen (TN) by the Kjeldahl method. NH_4_^+^-N and NO_3_^−^-N were extracted with 2 M KCl and determined by indophenol blue colorimetry and dual wavelength UV spectrophotometry (Varma et al., 2018); Using a conductivity meter and pH meter, the pH and EC were defined by a liquid-to-solid ratio of 10:1.

Based on molecular weight and functional carbon content, HS consists of two main components, humic acid (HA) and fulvic acid (FA). Typically, the TOC of humic components can characterize their concentration. Besides, the extraction and determination of humus was by the method of Zhou et al. (2014). Humus content was ascertained by mixing air-dried samples with an extract of 0.1 M Na_4_P_2_O_7_ mixed with NaOH at a solid–liquid ratio of 1:20 (w/v), shaking at 200 rpm for 24 h, and centrifuging at 12,000 rpm for 15 min at ambient temperature. FA was acquired by filtration through a 0.45 μm filter, and the precipitate was washed several times with 0.05 M HCl to acquire HA by dissolution with 0.05 M NaHCO_3_. The methodology utilized to determine total humic acid (HA) and fulvic acid (FA) was the externally heated potassium dichromate-volumetric method. Moreover, the total carbon content of the compost samples was determined and subtracted from the humic and fulvic acid content to obtain the humin content. The humification index was calculated based on the following formula (Wu et al., 2017b):


$$HA/FA=\frac{C_{HA}}{C_{FA}}$$

$$HI\;\left(\%\right)=\frac{C_{HA}}{TOC}\times 100$$

$$HPA\;\left(\%\right)=\frac{C_{HA}}{C_{HS}}\times 100$$

(Note: C_HA_, C_FA_, and C_HS_, indicate the TOC content of HA, FA, and HS; HA/FA represents Degree of Polymerization, HI represents Humification Index, and HPA denotes the Percentage of HA.)

The identification of humus precursors predominantly entails the analysis of sugar constituents, including polysaccharides, total sugars, reducing sugars, and polyphenols. Besides, total sugars were ascertained by the phenol-sulfuric acid method, reducing sugars were determined by the 3,5-dinitro salicylic acid (DNS) method, with polysaccharide content equal to total sugars minus reducing sugars, and polyphenols were ascertained by the FoLin–CiocaLteu (FC) (Singleton and Rossi, 1965).To each sample, add 800 μL of Na_2_CO_3_ (7.5% w,v), mix well, and allow it to rest for 2 min. Subsequently, add 1 mL of FC reagent. It was placed at 20°C for 2 h and the absorbance was measured spectrophotometrically at 765 nm.

### 2.3 DNA extraction and high-throughput sequencing

Samples from 1, 3, 17, and 36d were selected for high-throughput sequencing. DNA was extracted using a soil DNA kit (Phygene, Fujian, China) following the instructions, and before high-throughput sequencing on the Illumina MiSeq300 platform manufactured by Beijing Allwegene Technology, the extracted DNA underwent purification and amplification. Moreover, the fungal ITS sequence was determined by amplifying the ITS1 region with primers ITS1-F (CTTGGTCATTTAGAGGAAGTAA) and ITS2 (TGCGTTCTTCATCGATGC). The bacterial 16S sequences were determined by amplifying the V3-V4 region with primers 338F and 806R, and the raw data was retained after downloading in Fastq format. To preserve and share high-throughput sequencing data, the NCBI Data Center established the Sequence Read Archive (SRA) database.^1^ The raw data were processed through sequence splicing to remove low-quality sequences, followed by OTU clustering and species identification. In addition to species annotation results and alpha and beta diversity derived from OTU clustering outcomes, the analysis also incorporated variations in microbial community structure.

### 2.4 Statistical analysis

GraphPad Prism 9 was employed to analyze the physicochemical parameters, concentrations of humus precursors, and humus components. The IBM SPSS Statistics 22 software was utilized to perform every statistical analysis. The OTU table’s basic statistics, including the results of data normalization and the combination of species from distinct phyla, are calculated using the “dplyr” package of R.Alpha diversity was calculated utilizing the ‘vegan’ package and analyzed and plotted with the ‘EasyStat’ package. The beta diversity was analyzed and plotted using the R package “phyloseq” and “ggplot,” respectively. Computation of dissimilarity based on Bray-Curtis distances between samples to assess distinctions in microbial community structure. The default selection for the species composition analysis consisted of the 10 most abundant OTUs. The data was cleaned utilizing ‘dplyr’ and visualized using ‘ggplot’. Any remaining species were integrated and labeled as ‘other’, and the resulting graphs were displayed as stacked histograms. Procrustes analysis was employed to determine whether there was a substantial whole correlation between microbial community structure and environmental factors. Furthermore, network analysis was performed using “ggClusterNet” with Gephi for calculations and mapping, with default relationship thresholds of *r* > 0.8 and *p* < 0.05. To discover and explore network relationships, the 200 microbes with the highest abundance were chosen. The colors filled in on the network graphs represent taxonomic information at the phylum level. Gephi is utilized to visualize and compute topological attributes, including the number of nodes, edges, average degree, average path length, network diameter, as well as network density, to estimate the complexity of the network. Moreover, the stability of the network is evaluated through robustness analysis. Core microbes were identified by adopting the “plot(g)” package of R. The top 20 genera with the highest abundance in the network were selected and defined as core microbes. STAMP was employed to analyze the distinction between groups, and nonparametric tests were integrated with horizontal histogram visualization. Furthermore, *p*-value correction was performed by default utilizing “fdr” with a threshold of 0.05.RDA was performed utilizing Canoco (Version 5.0) to investigate the correlation between fundamental microbes and environmental variables. Structural equation modeling using AMOS (IBM; SPSS AMOS 20.0.0) was employed to assess the direct and indirect influences on the humus of core microbes and precursors.

## 3 Results and discussion

### 3.1 Changes in composting temperature

**Figure 1:**
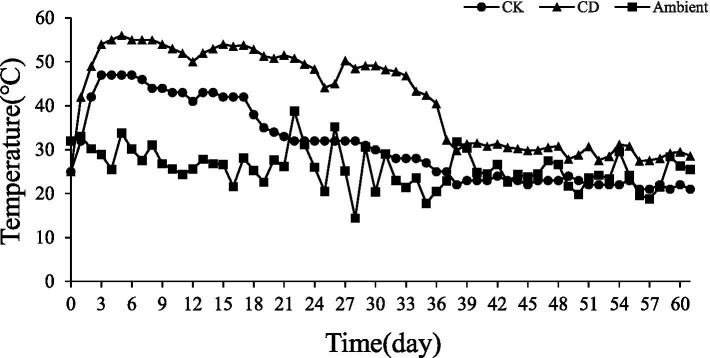
Changes in composting temperature.

The changes in pile temperature and ambient temperature during the composting process are shown (Figure 1). The pile temperature of all treatments increased rapidly in the first 3 d. The pile temperature of CD treatment reached 54°C in the 3rd d and rose to the highest temperature (56°C) in the 5th d. The whole high-temperature stage (>50°C) lasted for a total of 20 d. The temperature of the heap reached a peak in the 3rd d (47°C) after the CK treatment and was maintained for 4 days but the temperature never exceeded 50°C. The temperature of the compost pile after the CK treatment reached a peak in the 3rd d (47°C) and remained at the highest temperature for 4 days but the temperature never exceeded 50°C. The growth and development of microorganisms may have been hampered by the lack of effective carbon and nitrogen sources, which contributed to the low pile temperature. The heat generated by microbial decomposition is the main factor contributing to the temperature change of the heap, and the addition of cow dung to *Agaricus bisporus* pomace compost can increase the temperature and microbial activity of the heap, thus accelerating the composting process.

### 3.2 Changes in organic components and humification processes during composting

**Figure 2:**
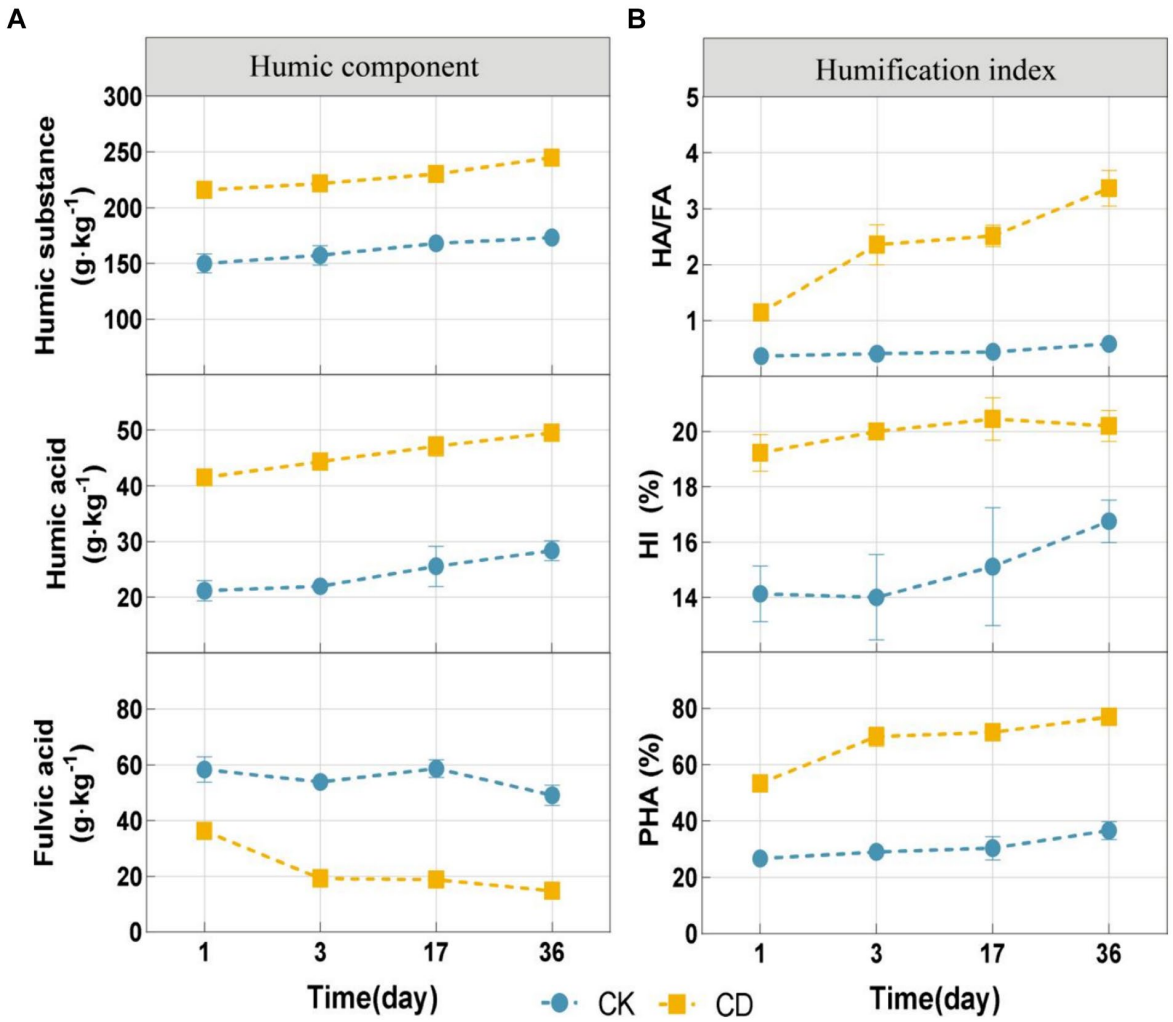
Changes in organic fractions and humification index during composting. **(A)** Humic components include humic substance, humic acid, and fulvic acid. **(B)** The humification index includes HA/FA, HI, and PHA.

To determine the effect of cattle manure addition on the humification process in the compost of *Agaricus bisporus* mushroom substrate, the fluctuations of humification indices and humic acid component concentrations (HA/FA, HI, and PHA) in the compost were analyzed. Besides, the contents of HA and HS indicated an increasing trend during composting (Figure 2A). Upon completion of the composting procedure, the contents of HA and HS in the composted material CD were higher (49.52 g·kg^−1^, 245.16 g·kg^−1^) than CK (28.33 g·kg^−1^,169.04 g·kg^−1^). On the contrary, the concentration of FA indicated a decreasing trend, with a 59.25% reduction in CD and a 15.82% reduction in CK. The humification process pertains to the metabolic activity of microbes on organic compounds. Throughout this cyclical process, interconversion between HA and FA is possible (Zhao et al., 2017a). Fulvic acid has a distinct structure characterized by low molecular weight and a high concentration of acidic functional groups. It is a comparatively mobile and active component of HS in compost, as opposed to HA (Tan, 2014). Due to its instability, fulvic acid is frequently transformed into more advanced aromatized humic substances, including HA (Zhou et al., 2014).In addition, microbes can utilize fulvic acid, a readily accessible organic compound, as an energy source (Zhang et al., 2018), thus resulting in an increase in HA concentration and a decrease in FA content.

The humification index, which accurately measures the degree of humification and maturity of the final compost, is calculated as the ratio of the HS, HA, and TOC indicators (Wei et al., 2014). HA/FA is widely used to describe the relative chelation rates of humic acid and fulvic acid and is frequently employed as a measure of the degree of compost humification (Zhao et al., 2017b). HI and PHA increases are indicative of an increase in the structural complexity of HS (Wu et al., 2017b). Under the experimental conditions, the composting process increased the levels of HA/FA, HI, and PHA in both treatments (Figure 2B). Throughout the composting process, the levels of HA/FA, HI, as well as PHA were substantially higher in the CD in comparison to the CK. These results indicate that the addition of cattle manure can remarkably enhance the humic acid conversion efficiency during the composting process and substantially promote the more mature humic acid components HA.

### 3.3 Changes in humus precursor concentrations during composting

**Figure 3:**
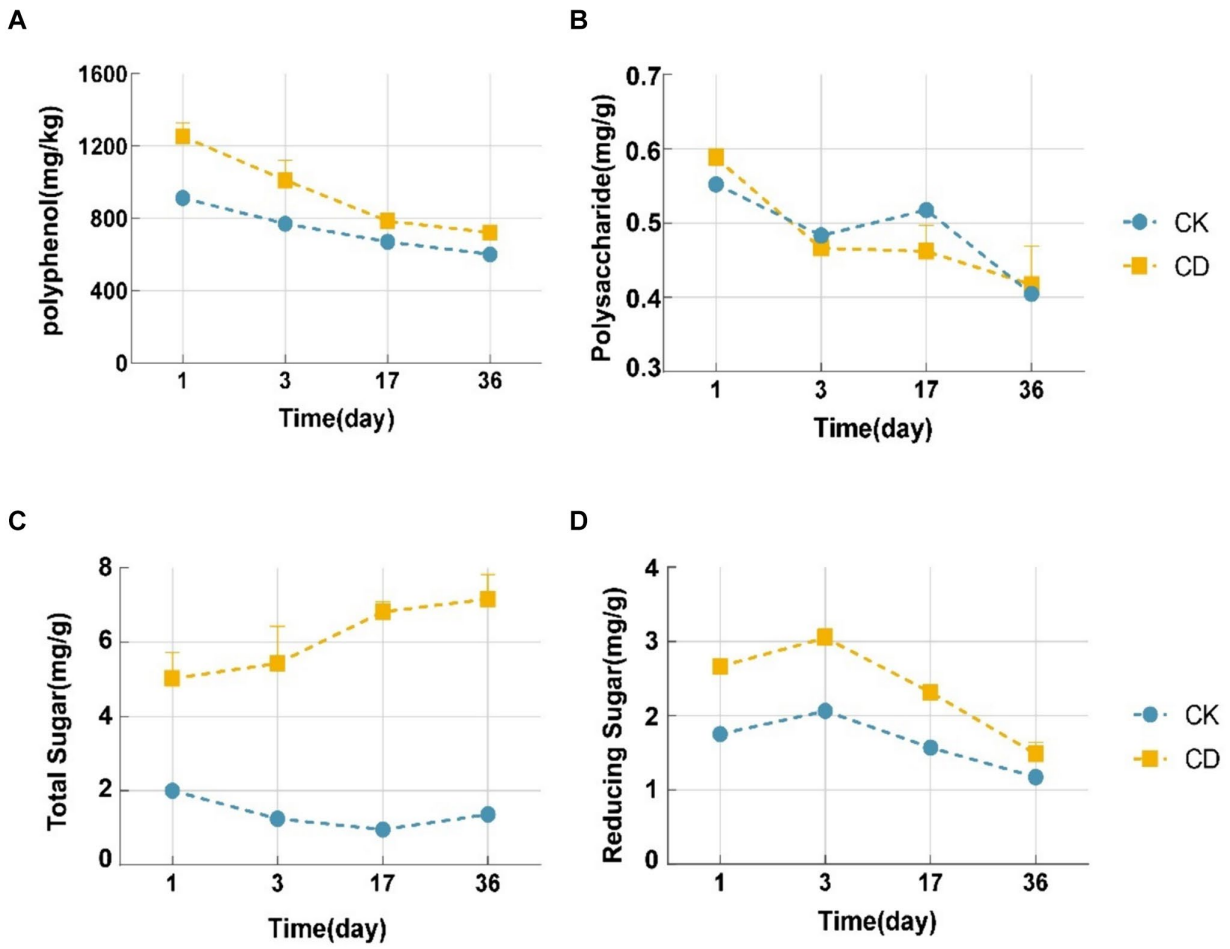
Changes in humus precursors during composting. **(A)** Polyphenol content. **(B)** Polysaccharide content. **(C)** Total sugar content. **(D)** Reducing sugar content.

Studies have demonstrated that the decomposition of organic matter during composting produces intermediate products including amino acids, polyphenols, polysaccharides, and reducing sugars that are strikingly similar to humus formation (Wu et al., 2020). The polyphenol content of the two treatments illustrated a decreasing trend as demonstrated in Figure 3A. The CD treatment exhibited a higher polyphenol content than the CK treatment at all time points. From 1d to 36d, the CD treatment witnessed a 531 mg/kg decrease in polyphenol content, whereas the CK treatment witnessed a 312 mg/kg decrease. Furthermore, the rate of decrease in the CD treatment was significantly greater than that of the CK. Under the action of polyphenol oxidase, polyphenols form benzoquinones, which subsequently undergo condensation reactions with intermediates including amino acids, polysaccharides, and reducing sugars (Duan et al., 2019b), which promotes the synthesis of humic substances, leading to a continuous decrease in polyphenol content. Substantial distinctions were observed between reducing sugars and polysaccharides during the initial 3 days of composting. The concentration of polysaccharides decreased slightly, and yet the concentration of reducing sugars increased during this period. This phenomenon could potentially be attributed to the degradation of polysaccharides to reducing sugars by microorganisms in response to the elevated compost temperature. There was an overall decreasing trend in polysaccharide concentration (Figure 3B), with a faster decrease in polysaccharide content in the CD treatment in comparison to CK, which was ascribed to the metabolic activities of microbes being stimulated by the addition of cattle manure. The two treatments illustrated opposite trends in total sugars, with the CK treatment initially decreasing and subsequently slowly increasing after the 17th day, while CD indicated an increasing trend in total sugars (Figure 3C). Reducing sugar functions as a carbon source for microorganisms and is utilizable directly by them. The concentration of reduced sugar indicated an increasing trend followed by a decreasing trend (Figure 3D). Both treatments reached their highest reducing sugar content on the 3rd day, with CD at 50.23% and CK at 45.31%. Nonetheless, the reduced sugar content of CD was constantly higher than that of CK. In the initial period, as the temperature increased, a gradual inactivation of certain mesophilic microbes accompanied a reduction in carbohydrate consumption, resulting in a slight increase in the concentration of reducing sugars on the 3rd day, which were subsequently directly utilized by the microbes, resulting in a rapid decrease. Consequently, we inferred that the reduction of polysaccharides and reducing sugars is related to microbial utilization and HS formation. Microbial metabolism is stimulated, the degradation of organic matter (including proteins and cellulose), and the synthesis of humus precursors are accelerated; the polymerization of additional carbohydrate components HS is encouraged.

### 3.4 Microbial community successions

#### 3.4.1 Analysis of microbial species composition and diversity

**Figure 4:**
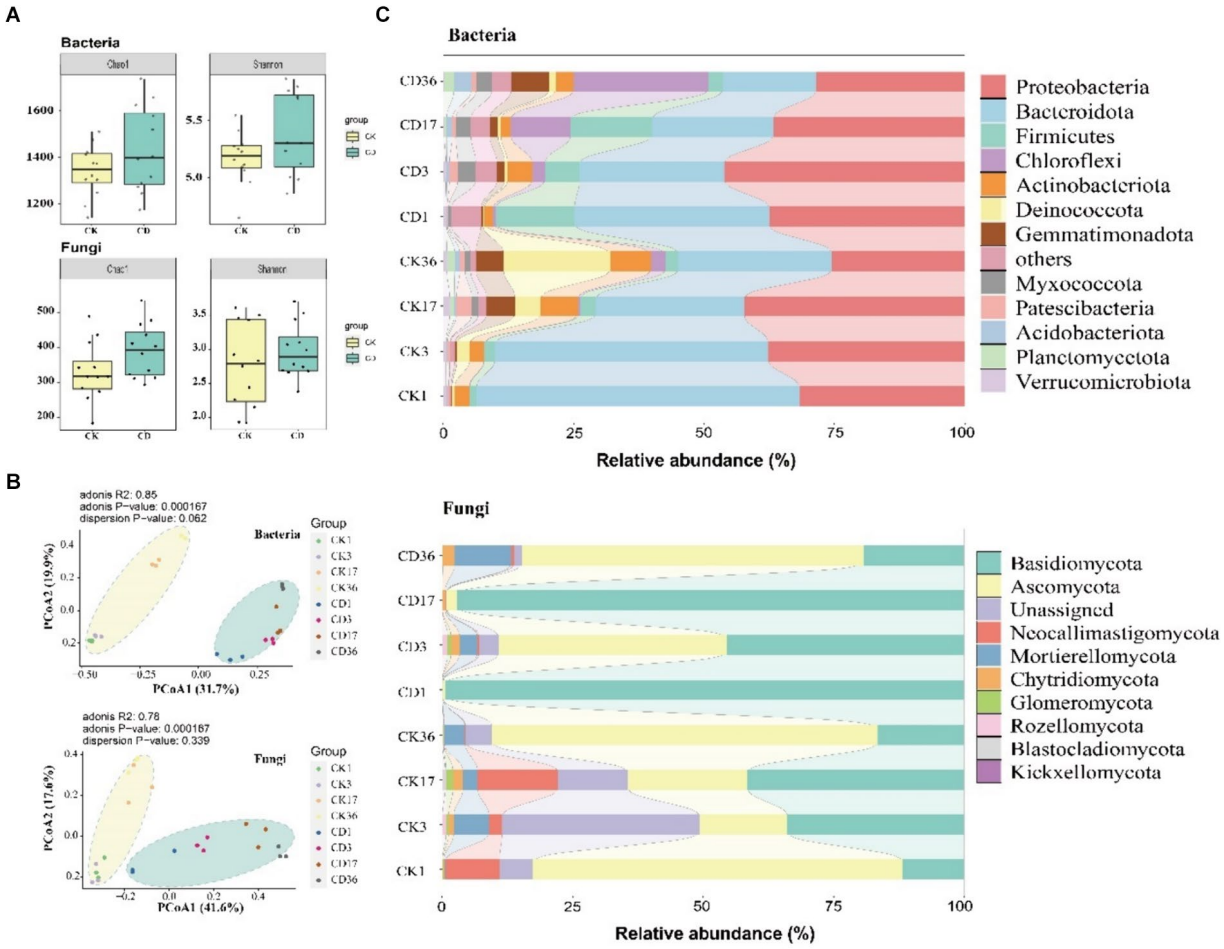
Microbial community composition and diversity in composting. **(A)** Bacterial fungal alpha diversity including Chao1 and Shannon indices. **(B)** PCoA demonstrates bacterial and fungal beta diversity. **(C)** Relative abundance of bacterial top13 and fungal top10 at the phylum level in the composting process.

Alpha diversity of bacteria and fungi was evaluated using Chao1 and Shannon’s index (Figure 4A). The diversity and abundance of the community were greater in CD than in CK. The transformation of organic components is more favorable on the condition that microbial communities are more diverse and abundant (Bello et al., 2020). The study investigated changes in bacterial and fungal community composition during composting via principal coordinate analysis (PCoA). Besides, PCoA 1 and PCoA 2 accounted for 31.7 and 19.9% of the total variance for bacteria, and 41.6 and 17.6% of the total variance for fungi (Figure 4B). Moreover, the two treatments were distinct into dual clusters with a tendency towards fragmentation. The composition of bacterial and fungal communities varied substantially among the distinct composting treatments, demonstrating that the addition of cattle manure influenced the succession of microbial communities. Meanwhile, previous studies have emphasized that chemical and organic components of manure have an important influence on the composition of composting bacterial communities (Li et al., 2017). Samiran examines the positive effect of nutrient addition on the overall microbial biomass and abundance of bacteria and fungi (Banerjee et al., 2016). The present study revealed that the microbial community in the mushroom substrate was predominantly impacted by the organic and chemical makeup of the animal manure.

To examine temporal changes in community composition more closely, we contrasted the modifications in the relative abundance of bacteria (Top13) and fungi (Top10) at the phylum level (Figure 4C). Besides, the bacteria consist mainly of *proteobacteria* and *Bacteroidota*. *Proteobacteria* are a substantial phylum of microbes that contain a wide range of metabolic species that can secrete numerous mixed enzymes that play an essential role in the degradation of organic matter. The data revealed an increasing trend followed by a decrease, with the CD reaching its peak value at 3d and the CK reaching its peak value at 17d before decreasing. *Bacteroidota* indicated a decreasing trend in all treatments, and *Bacteroidota* can degrade complex organic substances, including proteins and polysaccharides (Thomas et al., 2011). HA precursors may be present in these degraded small molecules during the final phase of composting. The most abundant fungal phylum are *Bacsidiomycota* and *Ascomycota*. They are the predominant fungi in organic matter degradation and can participate in the decomposition of inert carbon including lignocellulose (Yang et al., 2022). The abundance of *Ascomycetes* was higher in the early period of CD, particularly in the high-temperature phase, where it could utilize a variety of carbon sources and construct a unique anti-resistant structure that could survive in extreme conditions including high temperatures, and *Ascomycetes* was the dominant phylum in the final phase of composting.

#### 3.4.2 Relationships between microbial communities and environmental factors, indicators of humidity, and Maillard precursors

**Figure 5:**
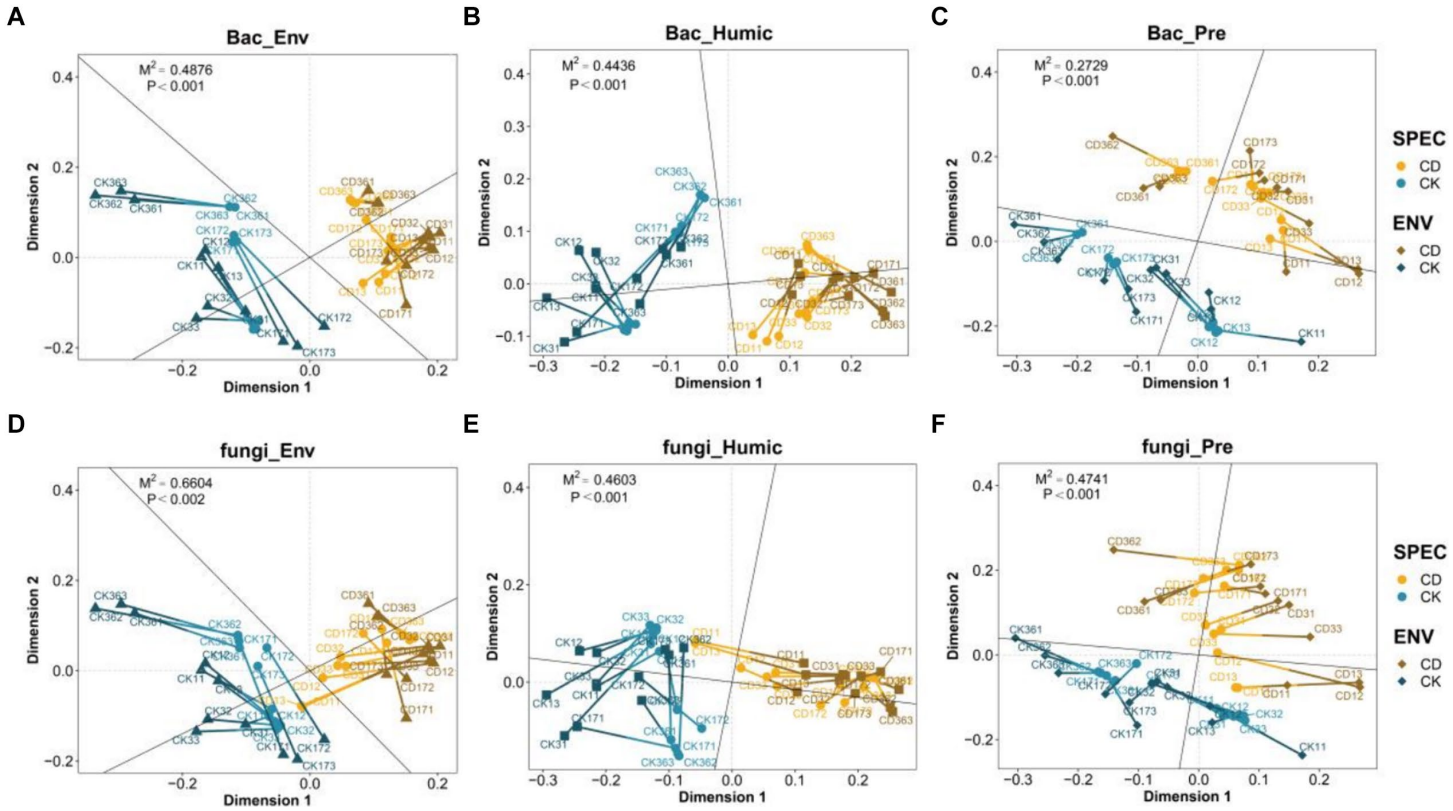
Procrustes analysis of the correlation among the Microbial community, physical and chemical indicators, based on the PCoA. Correlation between **(A)** bacteria and environmental factors **(B)** bacteria and Humic index. **(C)** Bacteria and humic precursor. **(D)** Fungi and environmental factors. **(E)** Fungi and Humic index. **(F)** Fungi and humic precursor. Each line segment in the graph represents a sample from a distinct group, with divergent colors indicating the groups. The points at either end of the line segment represent the microbiome data sample points and the environmental data sample points, respectively. The connecting line indicates the vector residuals of the two sorted configurations, which can evaluate the variation between the two datasets. The shorter the connecting line, the higher the consistency between the two datasets.

The overall potential consistency between bacterial fungal community abundance and environmental factors, humification indicators, and humus precursors was characterized using Procrustes analysis. M^2^ is used to assess the degree of correlation between the indicators, as well as a smaller M^2^ indicates a higher degree of correlation between the two datasets. Besides, environmental factor data including temperature, pH, EC, TOC, TN, NH_4_^+^-N, NO_3_^−^-N. (Detailed information is available in Supplementary Table S2). HS, HA, FA, HA/FA, HI, and PHA were included in the humic index data, while polyphenols, polysaccharides, reducing sugars, and total sugars comprised the humic precursor data. Procrustes’ analysis demonstrated that environmental factors, humification indicators, and humus precursors were substantially correlated with bacterial fungal communities (*p* < 0.001). As demonstrated, the degree of association between bacteria (Figures 5A–C) and other indicators (M^2^ = 0.4876, 0.4436, 0.2729) was greater than that of fungi (M^2^ = 0.6604, 0.4603, 0.4741) (Figures 5D,E). Studies have demonstrated that bacterial communities are more susceptible to environmental changes and abiotic influences than fungal communities (Yang and Wu, 2020). This is consistent with the findings of Yang and Wu (2020). In comparison to CK, the CD illustrated more clustered points and shorter connecting lines, suggesting that the correlation between the indicators and microbes is stronger.

#### 3.4.3 Microbial co-occurrence network analysis

**Figure 6:**
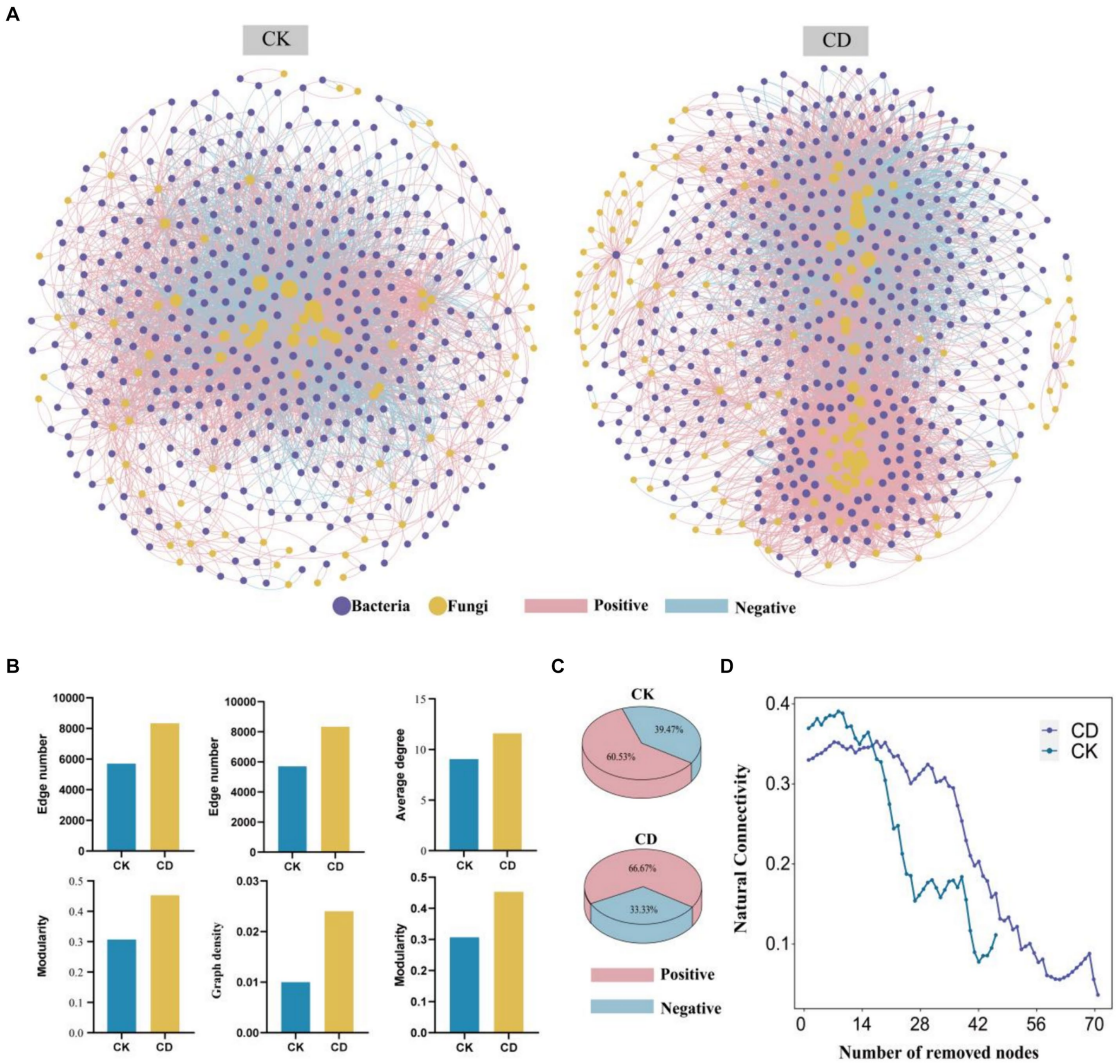
Complexity and Stability of Bacterial-Fungal Co-occurrence Networks. **(A)** Bacterial-fungal co-occurrence networks. **(B)** Key topological features of the network. **(C)** Percentage of positive and negative cohesion. **(D)** The robustness of the microbial networks is based on natural connectivity. The horizontal coordinate is the number of nodes removed and the vertical coordinate is the natural connectivity.

The transboundary co-occurrence network analysis to assess bacterial-fungal interactions between CD and CK. Besides, the co-occurrence network of CK consists of 492 nodes (bac 406, fun 86) and 5,706 edges, whereas CD consists of 590 nodes (bac463, fun 127) and 8,338 edges (Figure 6A). Key topological features of the network were employed to assess the complexity of soil microbial networks (Figure 6B). An expansion in the number of nodes, edges, average degree, network diameter, network density, and modularity coefficient results in an increase in the complexity of microbial networks (Ma et al., 2016). The results demonstrate substantial differences between the two treatments in co-occurring network topologies, with the CD network exhibiting more intricate connections. Additionally, the level of complexity of the network was evaluated by implementing a recently developed criterion for assessment termed “Cohesion.” Positive cohesion is defined as cooperative and reciprocal behavior between communities, whereas negative cohesion predominantly reflects competition for limited resources, unique environmental ecological niches, and spatial isolation (Yuan et al., 2021). The CD network exhibits a 66.67% higher positive cohesion than the CK network, which merely has a 60.53% positive cohesion (Figure 6C), indicating more (positive) reciprocal cooperation among microbes in the CD. The addition of cattle manure activates complementary effects between bacteria and fungi, which in turn can increase the metabolic capacity of the species. Previous studies have demonstrated that organic matter inputs can enhance the complexity of soil bacterial co-occurrence networks (Ling et al., 2016). The study determined that the increase in available nutrients after cattle manure application contributed to the changes in microbial function, enhanced bacterial-fungal interactions, and an increase in the complexity and correlation of microbial communities.

The performance and functionality of complex networks rely on their invulnerability or robustness, which is the ability to maintain connectivity even when some nodes are devastated or removed. The preservation of network invulnerability against destruction has emerged as a principal focus of research on complex networks, owing to its extensive array of applications. We modeled the resilience to node loss for co-occurrence networks to compute the robustness of the network (Figure 6D). Based on the natural connectivity analysis, the CD network has the highest robustness and more stable network. Moreover, the CK network has higher natural connectivity than the CD network on the condition that fewer nodes are removed randomly, and the CD has higher natural connectivity when more nodes are removed. The results of the study indicate that the microbial network became more stable and intricate with the addition of cow manure. This finding supports previous research, which illustrated that networks with higher complexity tend to be more stable (Yuan et al., 2021). The introduction of cattle manure influences the microbial environment, stimulates microbial metabolism, enhances the intricacy and stability of microbial networks spanning boundaries, triggers complementary interactions among bacteria and fungi, and reinforces collaboration and mutual support among microbes, which could be the primary factor behind the expedited process of humus formation.

#### 3.4.4 Relationships between core microbial community humic substances

**Figure 7:**
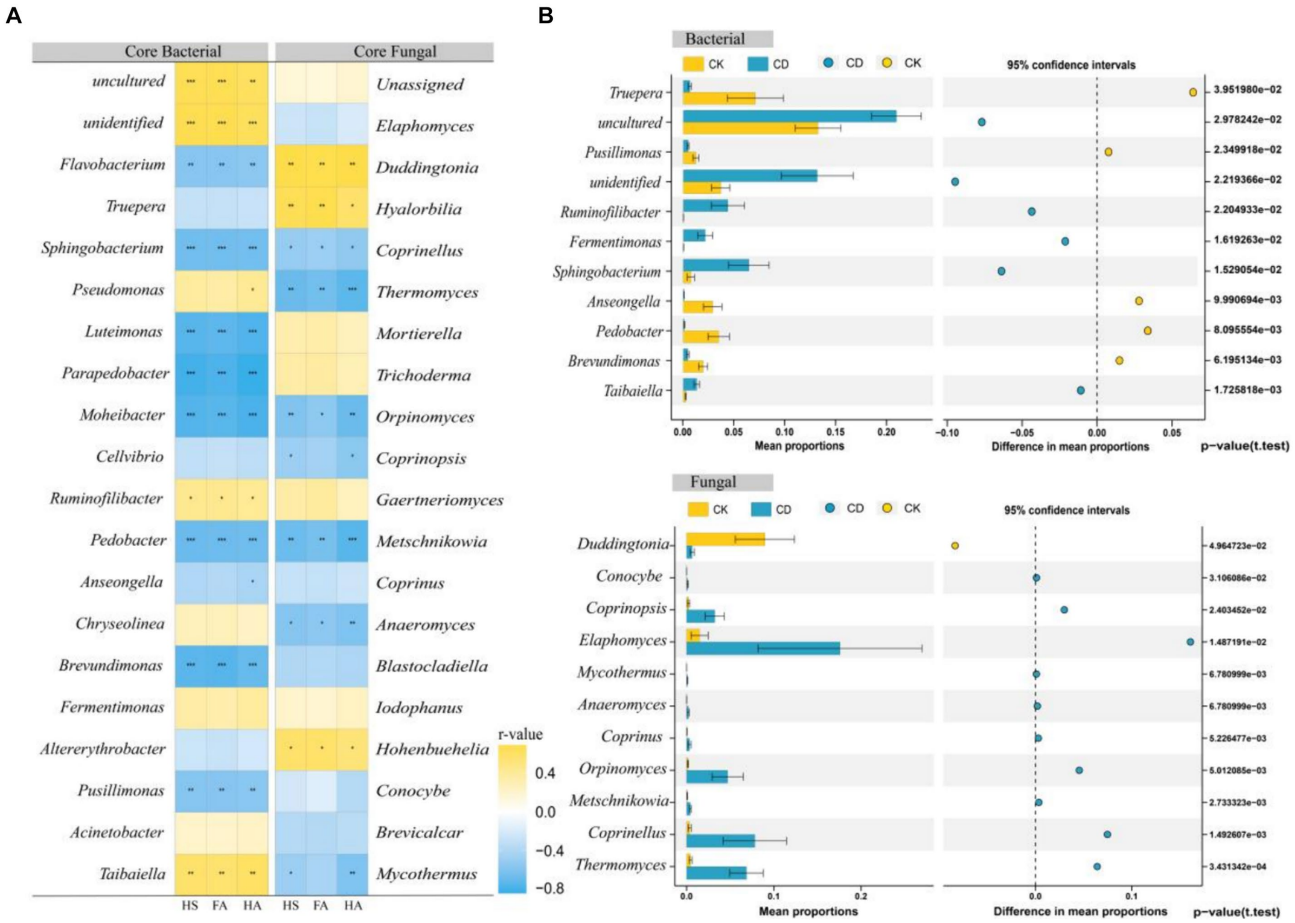
Core microbes and humus correlation analysis. **(A)** Heat map of Bacterial, fungi, and humus correlation. **(B)** Stamp analysis of core bacterial and core fungal.

The top 20 bacteria and fungi in the network in terms of abundance at the genus level were selected from the network and analyzed for correlation with each humification indicator utilizing Spearman’s correlation analysis (Figure 7A). There was a substantial positive correlation between the bacteria *Taibaiella*, *Ruminofilibacter*, *Fermentimonas*, *Uncultured*, *unidentified*, and fulvic acid, humic acid, humic substances. Conversely, *Flavobacterium*, *Sphingobacterium*, *Luteimonas*, *Parapedobacter*, *Moheibacter*, *Pedobacter*, *Brevundimonas*, and *Pusillimonas* indicated considerable negative correlations with each humification index. In regards to fungi, *Duddingtonia*, *Hyalorbilia*, and *Hohenbuehelia* indicated a positive correlation with fulvic acid, humic acid, as well as hummus. Conversely, however, *Coprinellus*, *Thermomyces*, *Orpinomyces*, *Metschnikowi*a, and others demonstrated a negative correlation with the humus indicator. Irrespective of whether the correlation is positive or negative, the core microbes are indispensable for humus formation. Positive correlations suggest that the microbes in question might be participating in the synthesis of humus components, while negative correlations suggest that they might have modified or employed humus components (Zhu et al., 2020), and thus these two groups of microbes are defined as processing microbes and transforming microbes (Qi et al., 2021).

STAMP analysis revealed considerable differences in 11 bacterial and 11 fungal genera between the two treatments. (Figure 7B) The CK demonstrated enrichment of 5 bacteria and 1 fungus, whereas the CD treatment indicated enrichment of 6 bacteria and 10 fungi. The majority of these core microbes with high abundance in CD are associated with humic acid formation. Research has demonstrated that *Ruminofilibacter*, a remarkable genus in anaerobic fermentation systems, performs an essential role in hemicellulose catabolism (Azman et al., 2015) and is linked to the formation of humic acid and carbon emissions (Yang et al., 2023).*Fermentimonas* help in the decomposition of organic matter and antibiotics that are challenging to break down (Zhao et al., 2022). *Taibaiella*, *Sphingobacterium*, and *Flavobacterium* are genera within the phylum *Bacteroidetes*. Studies have demonstrated that these genera aid in the progression of organic humification within compost. For instance, *Flavobacterium* is recognized to decompose large organic molecules including lipids and lignocellulose (Zhao et al., 2016; Mao et al., 2018). Furthermore, someone identified the genus *Taibaiella* as efficient in degrading aliphatic and aromatic hydrocarbon small molecules, including lignin structural units, short-chain starch, and organic acids (Diallo et al., 2021). Humus-polymerized macromolecules may be biodegraded via biotic and abiotic mechanisms (Zhang et al., 2019). *Coprinopsis* is derived from methylotrophic yeast and produces laccase, an enzyme that can degrade various organic chemical pollutants, illustrating chlorophenols and polycyclic aromatic hydrocarbons (PAHs) (Garcia-Delgado et al., 2015). Additionally, it promotes the oxidation of lignin and phenolics that led to the formation of HS macromolecules (Achtnich et al., 1999). *Coprinellus* facilitates the conversion of macromolecules, including dibenzothiophene (Aranda et al., 2009). *Thermomyces* is a thermophilic cellulolytic ascomycete that produces heat-resistant xylanases (Singh et al., 2003), which are essential in the degradation of polymeric carbohydrates. It has been isolated from compost and secretes over 60 distinct cellulases, hemicellulases, and other glycosyl hydrolases (Basotra et al., 2016). *Trichoderma* contributes significantly to cellulose decomposition (Strakowska et al., 2014). By stimulating the production of cellulose hydrolases, it facilitates the hydrolysis of cellulose and additionally encourages glycosaminic condensation reactions (Zhao et al., 2017c). Some white-rotting fungi, including *Coprinus*, have been determined to change the physicochemical properties of HA, resulting in decolorization, depolymerization, and mineralization effects on HS (Dehorter and Blondeau, 1992). Thus, the increased humification as an outcome of CD might be ascribed to the enrichment of this genus that can synthesize precursors from lignocellulose decomposition to humic substances. Interestingly, the results of the stamp distinction analysis illustrated that the bacteria enriched in CD were primarily processing microbes, while the fungi were mainly transforming microbes. It is assumed that bacteria may contribute more directly to the development of humus components, whereas fungi may be more involved in the further formation of HS through the transformation of precursor substances.

### 3.5 Possible mechanisms affecting humus formation

**Figure 8:**
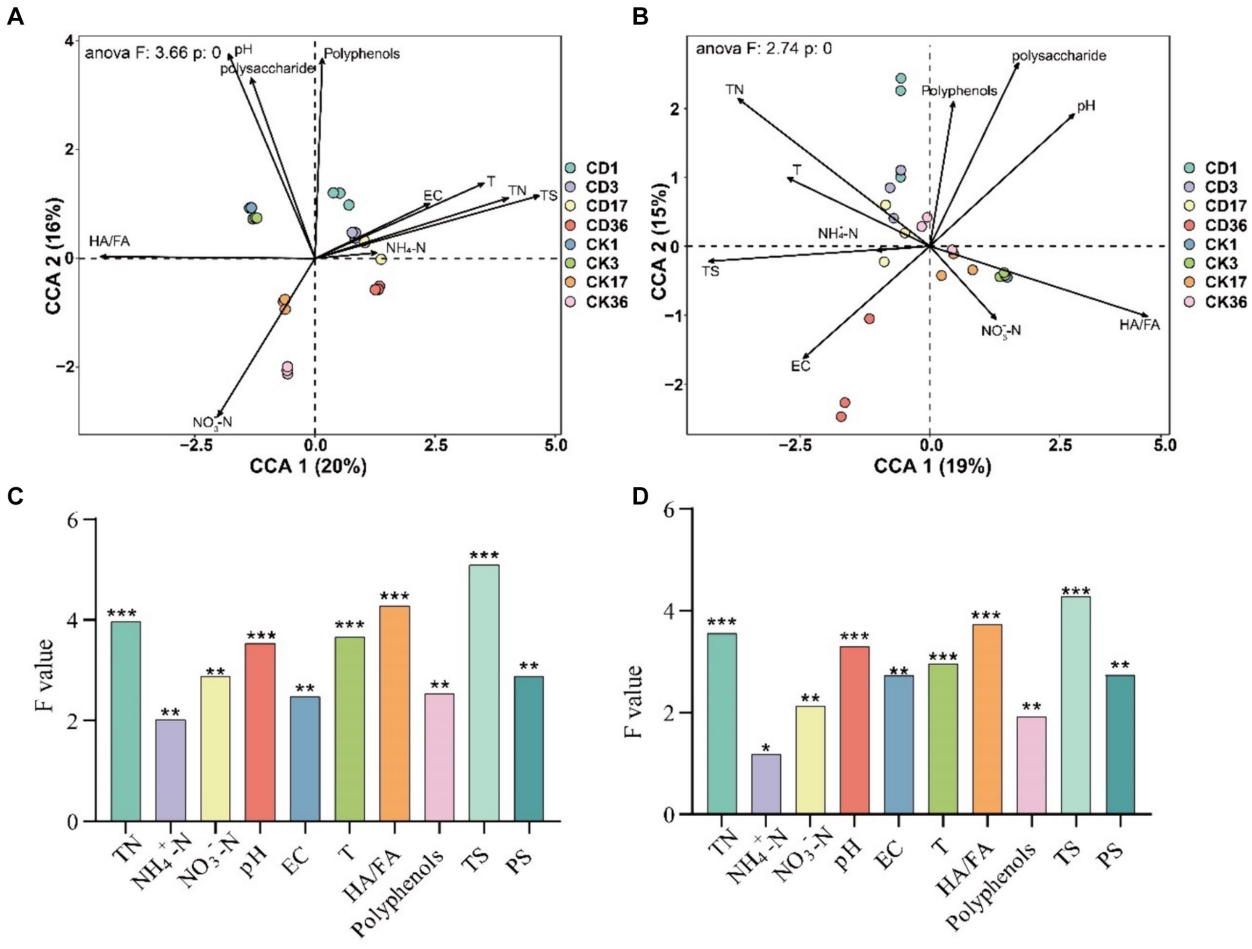
RDA analysis between environmental factors, humus indicators, and bacterial fungi. **(A)** Bacteria. **(B)** Fungi RDA analysis. The diagram displays environmental factors with black arrows and microbial sample groupings with colored circles. The arrow indicates the positive or negative correlation of the environmental factor with the ordination axis, and the length of the ray represents the degree of correlation between an environmental factor and the distribution of the community species. Longer ray extensions indicate greater correlation and vice versa. The size of the correlation between the indicators is represented by the angle between the rays. A smaller angle indicates a higher correlation, while a larger angle indicates a lower correlation. **(C)** Contribution of individual factors to the bacterial community. **(D)** Contribution of individual factors to the bacterial fungal community. TN, Total nitrogen; EC, Electrical conductivity; T, Temperature; HA/FA, Ratio of humic acid to fulvic acid; TS, Total Sugar; PS, Polysaccharide. Significance level: ^*^p < 0.05, ^**^p < 0.01, ^***^p < 0.001.

Physicochemical parameters exert an influence on the production of humic precursors, subsequently impacting microbial activity (Wu et al., 2017b).RDA was used to analyze the connection between physicochemical indicators, humus formation, bacterial (Figure 8A), and fungal (Figure 8B) activity during the composting process. Additionally, CCA was performed for non-restrictive sorting and the contribution of each environmental factor to the microbial community was calculated using anova, and the cca for significance testing (Figures 8C,D). Setting a threshold of *p* < 0.05 screened 10 indicators for correlation with microbes, with TS, N, T, pH, and HA/FA having the greatest effect on bacteria and fungi (*p* < 0.001). Overall, the indicators contributed more to the bacteria, and yet the difference was not remarkable. Polysaccharide is the primary precursor that affects the degree of humification, which is strikingly similar to HA/FA. The three most critical environmental factors affecting HA/FA are NO_3_^−^, pH, and N. Among these factors, NO_3_^−^ and pH are positively correlated with HA/FA, while N is negatively correlated with HA/FA. Additionally, T, EC, and NH_4_^+^ are negatively correlated with HA/FA.

**Figure 9:**
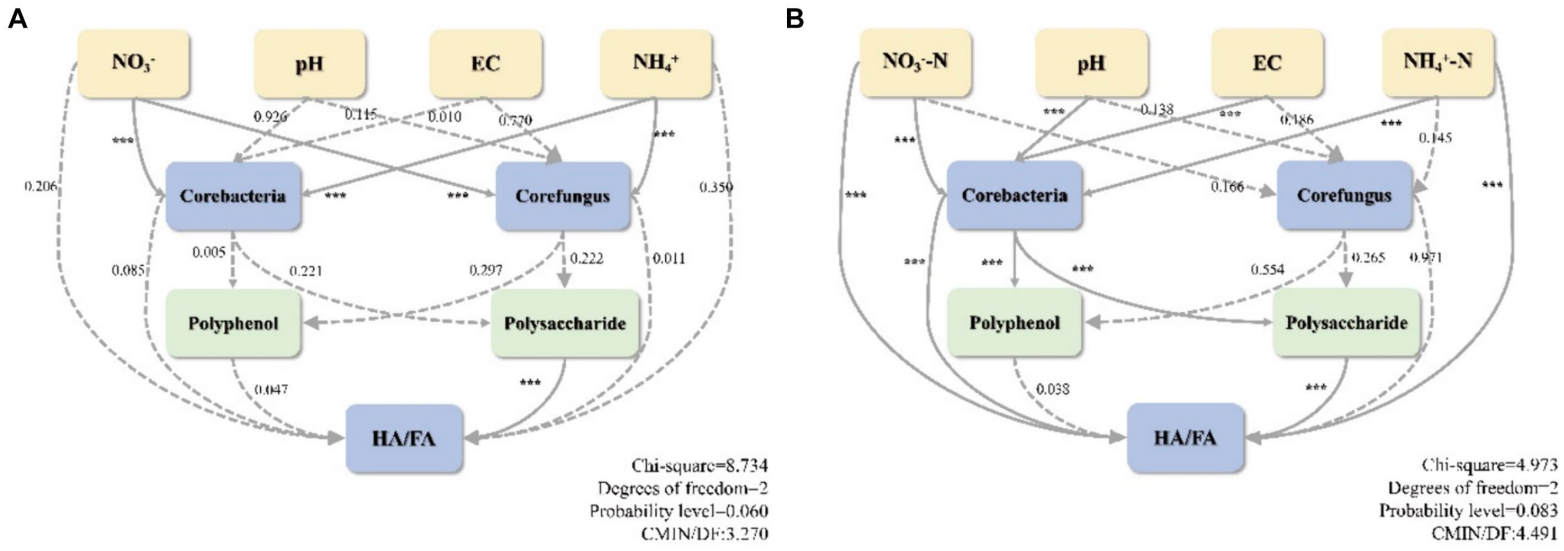
Structural equation modeling humification. **(A)** CK, **(B)** CD. The model’s variables are represented by boxes. Solid lines indicate considerable correlations, while dashed lines indicate substantial correlations. Correlation coefficients are denoted by numbers on the lines. Significance level: ^*^p < 0.05, ^**^p < 0.01, ^***^p < 0.001.

Structural equation modeling (SEM) was employed to validate the response of environmental factors, core microbes, as well as precursors to the humification index HA/FA and to evaluate the effect of cattle manure addition on the humification process (Figure 9). The correlation strength between HA/FA in RDA and humus formation led to the identification of three environmental parameters that influence humus formation: NO_3_^−^, pH, and NH_4_^+^. Additionally, polyphenols and polysaccharides were identified as essential precursors affecting humus formation, while core bacteria and core fungi were identified as drivers. Besides, SEM illustrated that the inclusion of cattle manure modified the correlation between physicochemical parameters and core microbes during the composting process. Particularly, the correlation between pH, EC, and the correlation between core bacteria and CD was reversed from positive to negative in CD relative to CK. Meanwhile, the correlation between NO_3_^−^ and core bacteria was strengthened yet weakened with core fungus. Additionally, the positive and negative correlations shifted. In SEM, a positive correlation signifies that two variables contribute to one another’s improvement; conversely, a negative correlation suggests that the variables are utilized or formed in opposition to one another. After the addition of cattle manure, the function of the core bacteria shifted, and the correlation with polyphenols and polysaccharides changed from a positive correlation to a negative correlation, implying that the function shifted from facilitating the production of precursors to transforming them directly into precursors. This is because polyphenols and polysaccharides are used by microbes as the predominantly energy and carbon source to facilitate the conversion of FA to HA (Wu et al., 2017b). Furthermore, the influence of CD core microbes on humus precursors (*p* < 0.001), which in turn relates to the extent of compost humification through polyphenols and polysaccharides, was increased in comparison to CK. Concurrently, the addition of cattle manure enhanced the direct impact of core bacteria on HA/FA. Confirming the previous assumption that bacteria are more directly involved in the formation of humus components, fungi continue to form HS through the transformation of precursors. The SEM analysis revealed a highly substantial negative correlation between polysaccharides and HA/FA in both treatments, which suggests that the primary precursors for the polymerization-mediated formation of HA are polysaccharides. Additionally, the results suggest that core bacteria play a more remarkable role than fungi in precursor production and humus formation. The SEM analysis indicated a highly considerable negative correlation between polysaccharides and HA/FA in both treatments, demonstrating that polysaccharides are the main precursors for the formation of HA through polymerization. Additionally, they concluded that core bacteria exert a more substantial influence on precursor production and humus formation than fungi.

## 4 Conclusion

In this study, the addition of cattle manure promoted the formation of humus components as well as accelerated the synthesis and decomposition rate of precursors including polysaccharides, polyphenols, and reducing sugars. This acceleration favored the formation of humic acids. Enhanced intricacy and resilience of microbial networks across boundaries, heightened collaboration and mutual support among microorganisms, and augmented prevalence of essential microbes engaged in the formation of humus. It increases the complexity and stability of co-occurrence microbial networks, enhances cooperative interactions among microbes, as well as increases the relative abundance of key microbes involved in humus formation. Moreover, the addition of cattle manure manipulated the correlation between microbial communities and physicochemical parameters, as well as humus formation during composting. The HS precursors generated by the microbial community’s synergistic action could potentially accelerate the humification process. Polysaccharides play a crucial role in the humification pathway by promoting the formation of humic acid. This conversion process results in the transformation of the unstable FA into the more mature HA, ultimately increasing the HA/FA ratio. Additionally, core bacteria including *Ruminofilibacter*, *Taibaiella*, and *Sphingobacterium* play a central role in material transformation and humus formation. These findings provide new ideas for regulating the degree of humification in the composting process and have important practical implications for optimizing mushroom cultivation and composting techniques today.

## Data availability statement

All data generated or analyzed during this study are included in the article. Also, all the raw sequences were deposited in the NCBI sequence read archive (SRA) under BioProject PRJNA1062035, https://www.ncbi.nlm.nih.gov/bioproject/PRJNA1062035.

## Author contributions

FY: Conceptualization, Funding acquisition, Investigation, Project administration, Resources, Supervision, Validation, Writing – review & editing. MW: Conceptualization, Data curation, Methodology, Software, Visualization, Writing – original draft, Writing – review & editing. LZ: Conceptualization, Formal analysis, Investigation, Methodology, Project administration, Supervision, Validation, Writing – review & editing. BF: Conceptualization, Formal analysis, Investigation, Project administration, Software, Supervision, Validation, Writing – review & editing. NS: Conceptualization, Data curation, Formal analysis, Investigation, Methodology, Resources, Software, Visualization, Writing – review & editing. JL: Data curation, Methodology, Software, Writing – review & editing. XS: Data curation, Methodology, Software, Writing – review & editing. ZD: Data curation, Methodology, Software, Writing – review & editing.

## Footnotes

^1^ http://www.ncbi.nlm.nih.gov/Traces/sra
